# Polymorphism in Cytochrome P450 3A4 Is Ethnicity Related

**DOI:** 10.3389/fgene.2019.00224

**Published:** 2019-03-19

**Authors:** Yelena Guttman, Adi Nudel, Zohar Kerem

**Affiliations:** ^1^Institute of Biochemistry, Food Science and Nutrition, The Robert H. Smith Faculty of Agriculture, Food and Environment, The Hebrew University of Jerusalem, Rehovot, Israel

**Keywords:** CYP3A4, ethnicity, polymorphism, food–drug interactions, nutrition, mutations, docking

## Abstract

Can mutations in Cytochrome P450 3A4 (CYP3A4), the major food- and drug-metabolizing enzyme, serve as biomarkers for personalized precise medicine? Classical genetic studies provide only limited data regarding the frequencies of CYP3A4 mutations and their role in food–drug interactions. Here, in an analysis of one large database of 141,456 individuals, we found 856 SNPs (single nucleotide polymorphism), of which 312 are missense mutations, far more than the previously reported dozens. Analyzing the data further, it is demonstrated that the frequency of mutations differs among ethnic groups. Hierarchical clustering divided the mutations to seven groups, each corresponding to a specific ethnicity. To the best of our knowledge this is the first comprehensive analysis of CYP3A4 allele frequencies in distinct ethnic groups. We suggest ethnicity based classification of CYP3A4 SNPs as the first step toward precise diet and medicine. Understanding which and when polymorphism might have clinical significance is a tremendously complex task. Using modeling approach, we could predict changes in the binding poses of ligands in the active site of single variants. These changes might imply clinical effects of the overlooked protein-altering CYP3A4 mutations, by modifying drug metabolism and FDI. It may be concluded that dietary habits, and hence FDI, are matters of ethnicity. Consequently, ethnic-related polymorphism in CYP3A4 and diet may be one underlying mechanism of response to medical regimes. The approaches presented here have the power to highlight mutations of clinical relevance in any gene of interest, thus to complement the arsenal of classic genetic screening tools.

## Introduction

For decades, food–drug interactions (FDI) and herb–drug interactions have been known to limit the success of medical treatments. The enormous number of possible interactions between genetic variations, medical regimes, and the numerous bioactive compounds found in food and herbs result in overwhelming complexity. Modern tools such as big-data analysis, machine learning, and simulation of protein–ligand interactions may help us to answer a whole set of questions: Might food choices contribute to the failure of therapeutic regimes and, if so, how? Which food(s) should be consumed prior to taking a prescribed drug? And probably the most exciting question: How can we use these tools to predict personal FDI? Clearly, many answers lie in the metabolism of drugs, foods, and herbs by cytochrome P450 3A4 (CYP3A4) in the liver and digestive tract (Galetin et al., 2010; Basheer and Kerem, 2015).

The majority of genes encoding CYP enzymes are polymorphic. To date, the most comprehensive source of information detailing CYP alleles is the Pharmacogene Variation Consortium^1^ [previously, the Human Cytochrome P450 (CYP) Allele Nomenclature Database], in which fewer than 100 alleles of CYP3A4 are represented. Of these, fewer than 40 are exonic SNPs (single nucleotide polymorphisms) that result in a modified protein sequence. The small number of subjects in all previously published works on CYP3A4 mutations provides us with limited data regarding true frequencies of CYP3A4 mutations in the whole population and in defined groups.

Not only that reliable information about SNPs incidence is incomplete, also their clinical implications are yet unclear in most cases (Zanger et al., 2014). Understanding which and when SNPs might have clinical significance is a tremendously complex task. *In vitro* assays are time-consuming, expensive and practically of low relevance considering the large amount of mutations and the endless number of food-drug combinations. Molecular-modeling methods, including docking and free-energy binding calculations, may serve to predict potential effects of SNPs and of many compounds on CYP3A4-mediated metabolism (Lewis et al., 1998). For instance, non-covalent, hydrophobic, electrostatic, and van der Waals interactions, all contribute to the orientation of a compound and hence to its binding and reacting at an enzyme’s active site. In turn, these will determine the enzyme’s affinity and specificity to different substrates, and the potency of enzyme inhibitors (Kirchmair et al., 2012; Basheer et al., 2017).

Here, we propose a new approach to measuring the allelic frequency of CYP3A4 mutations in different ethnic groups. This comprehensive approach has the power to highlight mutations that are prevalent in particular ethnic groups, and combined with screening for interacting chemicals, e.g., inhibitors from food will allow the elucidation of the effects of particular mutations on drug–food interaction, serving as an initial step toward personalized medicine and nutrition. This work may raise awareness of the possible clinical importance of protein-altering CYP3A4 SNPs and also suggests a few necessary tools for the promotion and application of precision and personalized medicine.

## Materials and Methods

### Database Screening and Data Analysis

The CYP3A4 variants dataset was downloaded from the gnomAD browser^2^ as a CVS file. Python 2.7 with NumPy, pandas and matplotlib packages was used for data analysis and visualization (see Supplementary Data Sheet S1). Agglomerative hierarchical clustering was performed using the Expander 7 software (Shamir et al., 2005) with the Pearson rank correlation coefficient as a measure of similarities and complete linkage type. A distance threshold of 0.6 was set for grouping of SNPs.

### *In silico* Polymorphism Modeling

Maestro 2017-2 release (Schrodinger, New York, NY, United States) was used for the computational modeling. CYP3A4 docking model was built as previously described (Basheer et al., 2017). In brief, CYP3A4 crystal structure (PDB entry 2V0M) was processed, modified and refined following the Protein Preparation Wizard steps. A docking grid with a metal coordination constraint for the Fe^2+^ in the heme group was generated based on the centroid of ketoconazole in the original binding site in the crystal structure. Seven mutations were selected for docking simulations, one as a representative for each ethnic group (Tables 1, 2). For each variant protein, a single point mutation was introduced prior to protein preparation steps. 3D structures of ligands were generated based on 2D structures from PubChem^3^ and prepared for docking using LigPrep task. OPLS3 force field and default Glide options for standard precision were applied for the docking model, with the exception that the metal coordination constraint was used, as well as 30 poses for the number of poses to include and 10 poses for the number of poses to write out. For each ligand, the docking result with the lowest Glide emodel score was selected.

**Table 1:** Selected representative SNPs for seven ethnic groups.

	Position	rsID	SNP	Amino acid change	Allele frequency	
					Total population	Ethnic group
European (non-Finnish)	99367392	rs4986908	C→G	D174H	1.97E-03	3.22E-03
European (Finnish)	99365983	rs55785340	A→G	S222P	1.04E-03	9.72E-03
Ashkenazi Jewish	99367771	rs748236460	T→C	T136A	2.05E-04	2.22E-03
Latino	99364768	rs199908125	C→T	E262K	1.26E-04	7.82E-04
African	99359800	rs12721629	G→A	L373F	3.47E-04	3.52E-03
East Asian	99361626	rs28371759	A→G	L293P	1.55E-03	1.92E-02
South Asian	99367408	rs568779023	C→G	K168N	3.42E-04	2.71E-03


**Table 2:** Frequency (%) of selected mutations by ethnic group.

	D174H	S222P	T136A	E262K	L373F	L293P	K168N
European (non-Finnish)	43.40	3.02	1.65	5.38	0	0	0
European (Finnish)	8.04	96.98	0	0	0	0	0
Ashkenazi Jewish	1.30	0	67.58	0	0	0	0
Latino	9.88	0	15.49	90.00	6.66	0.54	0
African	17.79	0	0	4.61	92.48	7.11	0
East Asian	0.67	0	15.28	0	0	92.04	0
South Asian	18.91	0	0	0	0.86	0.31	100.00


## Results

The Genome Aggregation Database (gnomAD; see text footnote 2) aggregates both exome- and genome-sequencing data from a wide variety of large-scale sequencing projects. It includes data from 125,748 exome sequences and 15,708 whole-genome sequences from 141,456 unrelated individuals representing seven ethnic populations (Lek et al., 2016). The GnomAD database presents 856 variants of CYP3A4, of which 397 are intronic and as many as 459 are exonic. Of the exonic SNPs, 312 are missense mutations, indicating that they affect protein structure. The CYP3A4 gene is 34,205 bp long. Its 13 exons comprise a 1,512-bp coding region that produces a protein of 504 amino acids. The 412 exonic SNPs with unique positions in this gene result in an exonic SNP density of 272/kbp (Supplementary Table S1).

**FIGURE 1:**
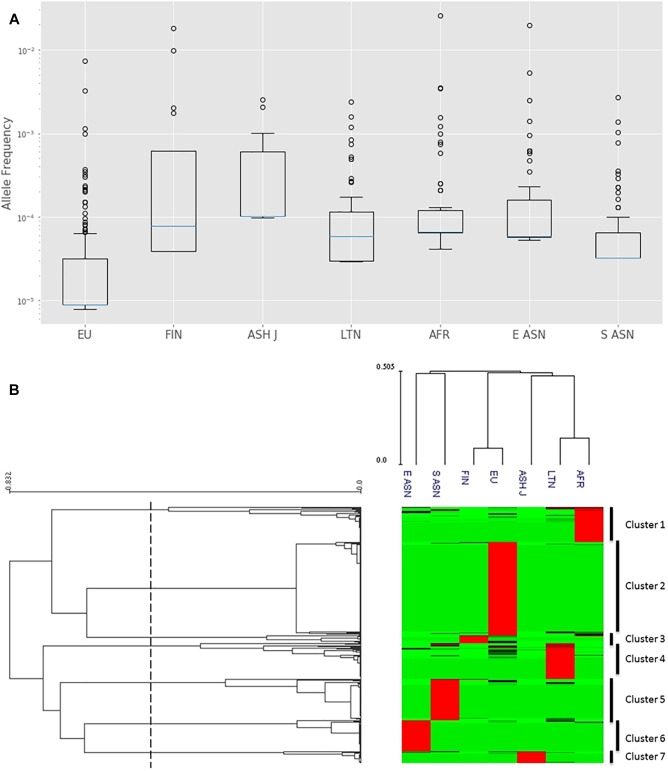
Analysis of CYP3A4 missense SNPs in seven distinct populations. **(A)** Log-scale box plot of allelic frequencies. Boxes represent the interquartile range (IQR), blue lines represent the medians, whiskers represent data within 1.5 IQR and outliers are shown as small circles. **(B)** Hierarchical clustering of allelic frequencies. Each row represents a single SNP. Each column represents distinct ethnic population. The allele frequency of the SNPs in each of the populations is represented by the color of the corresponding cell in the matrix file. Green and red represent low and high frequency, respectively. The upper dendrogram shows similarities in the allele frequency pattern between each group of subjects. The left dendrogram represents the clustering of genes in two groups. The dashed line represents the 0.6 distance threshold used for splitting to groups. EU – European (non-Finnish; *n* = 64,603), FIN – European (Finnish; *n* = 12,562), ASH J – Ashkenazi Jewish (*n* = 5,185), LTN – Latino (*n* = 17,720), AFR – African (*n* = 12,487), E ASN – East Asian (*n* = 9,977), S ASN – South Asian (*n* = 64,603).

Calculation of differential allele frequencies per ethnic group reveals that some populations exhibit higher frequencies of mutations (Figure 1A). Most of the CYP3A4 mutations in the European population are indeed rare, as is commonly thought, while mutations in other populations, such as African and East Asian, are much more prevalent (Supplementary Table S2).

We used hierarchical clustering to group variants with similar frequency patterns. Our data analysis yielded seven distinct clusters (Figure 1B). Further, it is clearly observed that high-frequency SNPs in each cluster are characteristic to one specific population. Hierarchical clustering analysis of the ethnic groups supports the association between genetic variance and ethnicity by grouping together related ethnicities such as South and East Asians as well as Finnish and non-Finnish Europeans.

A computational model was used to assess the possible influence of point mutations in CYP3A4 on its ability to bind substrates and inhibitors. CYP3A4 is able to oxidize a wide range of endogenous and xenobiotic compounds. Here, ketoconazole was selected as a representative drug and a very efficient specific inhibitor; androstenedione and testosterone were selected as representative endogenous hormone; and demethoxycurcumin and epigallocatechin were selected as representatives of dietary bioactives. A docking model was built to predict the binding poses of the selected compounds in the CYP3A4 binding site. The model was first validated by successfully restoring the ketoconazole pose in the binding site, with an RMSD of 1.52 Å relative to the original crystal structure. Seven mutant proteins were designed based on the crystal structure of the wild-type protein (Supplementary Figure S1). For each ethnic group, the most frequent unique mutation was selected as representative. The effect of single mutations on the substrate binding was assessed based on the comparison between docking poses onto the native protein and onto variant proteins. Changes in docking poses in terms of RMSD are summarized in Table 3.

**Table 3:** RMSD relative to the WT of docking ligands to binding sites of seven CYP3A4 variants.

	L293P	L373F	S222P	T136A	E262K	D174H	K168N
Ketoconazole	0.3443	1.6266	10.397*	0.3917	0.401	0.421	1.3963
Testosterone	0.0707	0.2396	0.2125	0.2189	0.2858	0.286	0.2467
Androstenedione	2.6064*	6.4981*	2.5886*	6.5049*	0.267	0.1832	0.1579
Demethoxycurcumin	0.017	0.1031	7.1577*	0.0286	0.0114	0.0081	0.0227
Epigallocatechin	0.0021	6.8436*	0.0108	0.404	0.4037	0.4032	0.4039


**FIGURE 2:**
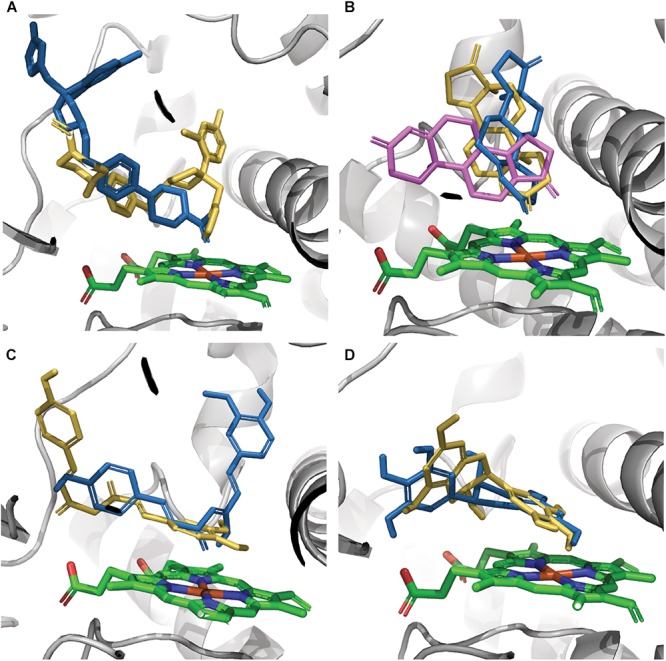
Models of ligands docked at the binding site of CYP3A4. **(A)** Ketoconazole, **(B)** androstenedione, **(C)** demethoxycurcumin, and **(D)** epigallocatechin. The protein-binding site is represented by gray ribbons; heme is represented by green sticks, docking poses in the WT protein and in S222P and L373F mutants are shown as orange, blue, and violet sticks, respectively. Androstenedione docking poses in L293P and in T136A variants overlap the poses in S222P and in L373F variants, respectively.

*Poses marked with ^∗^ are presented in Figure 2.*

The effect of CYP3A4 SNP on substrate binding was found to be mutation-substrate specific. Only in a few cases mutations caused a change in the binding pose of a ligand in the binding pocket. Testosterone docking pose was the same in all seven tested variants. The E262K, D174H, and K168N variants did not cause a binding pose change in any of the tested molecules. However, the L373F and T163A mutations changed the binding pose of androstenedione so that it was positioned parallel to the heme group rather than perpendicular to it, as in the WT protein. Also, androstenedione was rotated so that the cyclopentanone group is located proximal to the heme, instead of the cyclohexanone group in the WT protein. The S222P and L293P mutations caused only a small rotation in the binding pose of androstenedione (Figure 2A). Of all examined mutations, only S222P caused substantial changes in the docking poses of ketoconazole and demethoxycurcumin at the binding site (Figure 2B,C); whereas for epigallocatechin, the pose-changing mutation was L373F (Figure 2D).

## Discussion

Cytochrome P450 3A4 is the major enzyme responsible for food–drug interactions. Current research into mutations in CYP3A4 has been focused on a few dozen SNPs found in designated studies (Sata et al., 2000; Dai et al., 2001; Eiselt et al., 2001; Hsieh et al., 2001; Lamba et al., 2002; Murayama et al., 2002). As demonstrated here, they represent the tip of an iceberg considering the prevalence and potential outcomes of CYP3A4 mutations. The abundance of large-genome and exome-sequencing projects has opened a new avenue for the identification of many unknown mutations. Here, we show that the previously presented mutations are only the tip of the iceberg, by demonstrating 856 mutations existing in CYP3A4, of which one third modify the protein structure. Using a cohort of 141,456 unrelated individuals, accurate allelic frequencies of CYP3A4 mutations was calculated for seven separate ethnicities. To the best of our knowledge, this is the largest and most comprehensive large-data study of CYP3A4 exonic mutations and their allele frequencies in different populations, published to date.

Polymorphic CYP3A4 enzymes may be very important in explaining differences in drug efficacy and toxicity among different individuals. Mutations in the CYP3A4 gene might lead to abolished, reduced, altered or increased enzymatic activity. Exonic mutations can modify enzymatic activity, as has been demonstrated in a few clinical studies with selected substrates. Some cases of altered metabolism due to SNPs in CYP3A4 have already been described in the literature (Eiselt et al., 2001; Miyazaki et al., 2008). Despite the functional importance and clinical relevance of SNPs in CYP3A4 and possibly due to their relatively low identified frequency in the general population, polymorphism in CYP3A4 has not received the attention it deserves.

Here, seven mutations served to predict the effect of SNPs on substrate- and inhibitor-binding orientation. In the literature, CYP3A4 polymorphism divides the general population into three groups – poor metabolizers, normal metabolizers, and rapid metabolizers, based on intronic SNPs that modify expression levels rather than structure (Zanger and Schwab, 2013). Our calculations suggest an additional classification: the altered metabolizers. Some mutations proposed by our virtual model would cause a change in the binding orientation of individual ligands. These changes would be expected to decrease the probability of enzymatic oxidation due to increased distance from the heme, or lead to products that would otherwise not be evident during toxicity tests carried out as part of the drug-development process. However, as our model predicts, for most substrates CYP3A4 mutations are benign.

Modified position of a substrate in the binding pocket due to protein structural change is only one possible mechanism by which a mutation might change a protein’s activity. Impaired anchoring of the protein to the membrane, damaged substrate-leading channels, and compromised exit of the products present additional mechanisms for a mutational change in a protein’s activity. As shown here, the effect of every mutation is substrate-specific. Determining which combinations of substrates and mutations might modify the enzymatic activity, using traditional *in vitro* methods is laborious, emphasizing the need in predictive virtual tools in resolving this complex puzzle.

Public and professional interest in personal and precision medicine is growing rapidly. Prediction of modified drug metabolism based on individual polymorphism in CYP3A4 seems to be only a matter of time. Here, we propose that distinct ethnic groups bear unique sets of CYP3A4 SNPs. Indeed, ethnicity may serve as a first feasible step in personalized medicine, preceding the implementation of an individual DNA screen for all. Interestingly, ethnicity has one more implication for CYP3A4 drug metabolism, being a major factor in determining food choices and dietary habits. It may be suggested that therapeutic regimes should be specifically designed for each ethnic group, at least for drugs that are highly metabolized by CYP3A4. This highlights the opportunities for harnessing and integrating databases and deep learning to identify how SNPs, ethnicity, dietary compounds and drugs modify CYP3A4 activity and the success of a medical regime.

## Data Availability

Publicly available datasets were analyzed in this study. This data can be found here: http://gnomad.broadinstitute.org/gene/ENSG00000160868.

## Author Contributions

All authors listed have made a substantial, direct and intellectual contribution to the work, and approved it for publication.

## Conflict of Interest Statement

The authors declare that the research was conducted in the absence of any commercial or financial relationships that could be construed as a potential conflict of interest.

## Supplementary Material

The Supplementary Material for this article can be found online at: https://www.frontiersin.org/articles/10.3389/fgene.2019.00224/full#supplementary-material

FIGURE S1 3D ribbon model of CYP3A4 and the location of the mutated amino acids in the seven variant proteins designed for docking. Heme is represented as green sticks, Fe^2+^ is represented as a red sphere, SNPs used in the *in silico* analysis are represented as red areas on the ribbon and R groups of mutated amino acids in variant models are shown explicitly as light gray sticks.

TABLE S1 CYP3A4 SNP types in a population of 141, 456 unrelated individuals representing 7 ethnic populations.

TABLE S2 CYP3A4 SNPs by ethnic group.

## Footnotes

1 www.pharmvar.org

2 https://gnomad.broadinstitute.org

3 https://pubchem.ncbi.nlm.nih.gov
