# Supplementary figures and images for: Polymorphism in Cytochrome P450 3A4 Is Ethnicity Related
Source: Front Genet. 2019 Mar 19;10:224. doi: 10.3389/fgene.2019.00224 (PMC6433705; doi:10.3389/fgene.2019.00224)

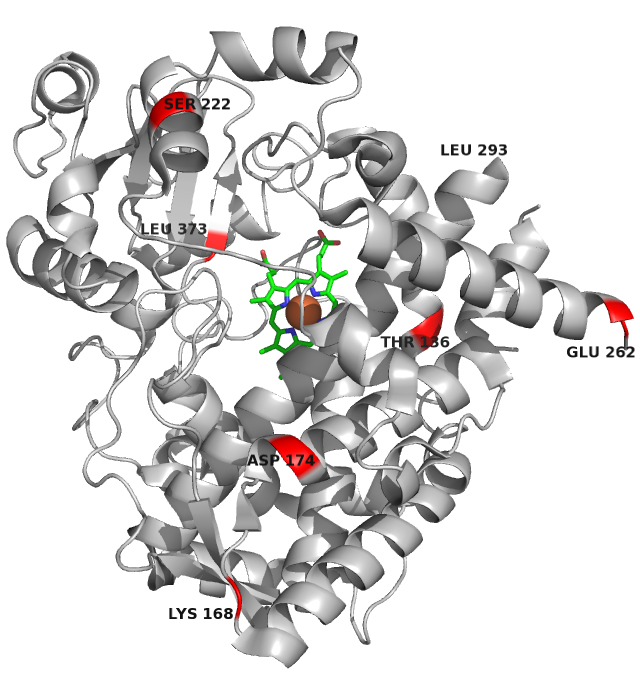

Supplement: FIGURE S1 — 3D ribbon model of CYP3A4 and the location of the mutated amino acids in the seven variant proteins designed for docking. Heme is represented as green sticks, Fe2+ is represented as a red sphere, SNPs used in the in silico analysis are represented as red areas on the ribbon and R groups of mutated amino acids in variant models are shown explicitly as light gray sticks. [file Image_1.png]
