# Supplementary material for: Polymorphism in Cytochrome P450 3A4 Is Ethnicity Related
Source: Front Genet. 2019 Mar 19;10:224. doi: 10.3389/fgene.2019.00224 (PMC6433705; doi:10.3389/fgene.2019.00224)
Supplement: TABLE S1 — CYP3A4 SNP types in a population of 141, 456 unrelated individuals representing 7 ethnic populations. [file Table_1.docx]

**Supplementary Table 1:** CYP3A4 SNP types in a population of 141,456 unrelated individuals representing 7 ethnic populations

|  | **SNPs** | **Uniquely positioned SNPs** |
| --- | --- | --- |
| **Intronic** | 397 | 352 |
| **Exonic** | 459 | 412 |
| Synonymous | 99 | 94 |
| Missense | 312 | 284 |
